# Role of umbilical cord C-peptide levels in early prediction of hypoglycemia in infants of diabetic mothers

**DOI:** 10.1186/s12887-021-02547-w

**Published:** 2021-02-17

**Authors:** Ahlam M. Saber, Magdy A. Mohamed, Abdelrahim A. Sadek, Ramadan A. Mahmoud

**Affiliations:** 1 Department of Pediatrics, Faculty of Medicine, Sohag University, 15 University Street, Sohag, 82524 Egypt; 2 Department of Obstetrics and Gynecology, Faculty of Medicine, Sohag University, Sohag, Egypt

**Keywords:** Infants of diabetic mothers, Umbilical cord C-peptide, Blood glucose, Full-term infant

## Abstract

**Background:**

Until now, diabetes during pregnancy has been associated with a high risk of maternal, fetal, and neonatal morbidities and mortalities. The main aim of this study was to evaluate the risk factors of hypoglycemia in infants of diabetic mothers (IDMs) and to study the relationship between umbilical cord (UC) C peptide levels and the risk of developing hypoglycemia.

**Material and methods:**

UC blood C-peptide and serial serum blood glucose measurements were done for all included singleton newborns born to diabetic mothers during the study period. Maternal and neonatal data such as gestational age, maternal age, maternal weight, types of diabetics and its control, maternal glycated hemoglobin (HbA1C), birth weight, Apgar score, and neonatal complete blood picture were collected.

**Results:**

In total, 83 IDMs met the inclusion criteria. Fifty-four (65.06%) developed hypoglycemia and 29 (34.94%) remained normoglycemic. However, there were no significant differences between hypoglycemic and normoglycemic IDMs in terms of types of maternal diabetics (*P* value = 0.41), its duration (P value = 0.43). The hypoglycemia peak occurred within the first 3 h of life, with 33.11 ± 8.84 mg/dl for the hypoglycemia group and 54.10 ± 6.66 mg/dl for the normoglycemic group (*P* value < 0.0001). Most of the babies had no hypoglycemic manifestation (96.30%). Neonates with hypoglycemia their mothers had poor diabetes control in the last trimester (HbA1C 7.09 ± 0.96%) compared to normoglycemic babies (HbA1C 6.11 ± 0.38%), (*P*-value < 0.0001). The mean (SD) of UC C-peptide level in hypoglycemic neonates increased to 1.73 ± 1.07 ng/ml compared to normoglycemic ones with 1.08 ± 0.81 ng/ml (*P* value = 0.005).

**Conclusion:**

Poor diabetes control, especially in the last trimester, is associated with neonatal hypoglycemia. Increased UC C-peptide levels could be used as an early indicator for the risk of developing neonatal hypoglycemia and a predictor for babies need neonatal admission.

## Introduction

Despite marked declines in neonatal mortality nowadays [1], diabetes mellitus (DM) with pregnancy either gestational (GDM), type 1, or 2 is still associated with a risk of maternal, fetal, and neonatal morbidities and mortalities. Moreover, its prevalence did not decline, as GDM was about 8.74% on one cohort [2]. Infants of a diabetic mother (IDM) often have complications closely linked to fetal hyperglycemia and hyperinsulinemia, induced by maternal hyperglycemia [3].

In the first trimester, maternal hyperglycemia can cause spontaneous abortions or major birth defects such as truncus arteriosus or aortic coarctation. In the second and third trimesters, maternal hyperglycemia can cause fetal hyperglycemia and hyperinsulinemia, which lead to post-natal neonatal hypoglycemia, hypocalcemia, polycythemia, hyperbilirubinemia, septal myocardial hypertrophy, delayed lung maturation, and macrosomia [4].

Most IDMs develop asymptomatic hypoglycemia in the first postnatal hours, as after delivery, the transplacental supply of high glucose is stopped. Hyperinsulinemic hypoglycemia is a major risk factor for brain injury and subsequent neurodevelopmental impairments; therefore, rapid identification and prompt management of the newborn with hypoglycemia are essential to avoid brain damage [5]. In this context, early detection of babies at high risk of hypoglycemia is important.

Human C-peptide is a 31-amino acid chain secreted from the beta cells of the pancreas in equimolar ratio with the insulin level. It was chosen over insulin to estimate neonatal hyperinsulinemia, as C-peptide has a long half-life and is unaffected by several blood processing conditions such as hemolysis [6, 7].

Maternal control during pregnancy mainly depends on diet and insulin control. The degree of control can be increased by serial measurements of blood glucose (BG) and glycated hemoglobin (HbA1C). However, HbA1C, now the current gold standard marker for glycemic control, reflects the BG level over the previous 2–3 months. It is a strong predictor of diabetic complications, and the cut-off used is 6.5% to diagnose diabetes [8].

Therefore, the main aim of this study is to evaluate the risk factors of hypoglycemia in IDM and its relation to maternal DM control in the last trimester. Furthermore, the relationship between UC C peptide and the risk of developing hypoglycemia was evaluated.

## Material and methods

### Design

The current clinical study was performed at the neonatal intensive care unit (NICU) in the Pediatrics Department, in cooperation with the Department of Obstetrics and Gynecology, Egypt, during the period from June 2018 to June 2019. Local ethical approval for the study was obtained from the Research Committee of the Faculty of Medicine at Sohag University (No. 321, 2018), and written informed consent was obtained from all parents of the children.

We included all singleton newborns born to diabetic mothers. Exclusion criteria included IDMs with preterm delivery, major congenital malformation at birth, severe perinatal asphyxia, twins, or erythroblastosis fetalis.

Eighty-three full-term singleton IDM newborns met the inclusion criteria and were enrolled in the study. The case group in this study consisted of any newborn infants delivered to DM mothers and who developed hypoglycemia within the first 24 h of life (BG less than 47 mg/dl), other IDMs maintaining normoglycemic during the study period served as controls. Both the cases and the controls groups were drawn from the same population characteristics.

Maternal data such as maternal age, gestational age, maternal weight, type and duration of DM, maternal drugs for the control of DM, maternal diseases such as pre-eclampsia, premature rupture of membranes (PROM), mode of delivery, and the presence of meconium in the amniotic fluid were recorded. Maternal HbA1C was performed. Neonatal data such as gender, neonatal weight, Apgar score at 1 min and at 5 min, causes of admission to NICU, if indicated, birth injuries, and detailed systemic examination were recorded. Observation for any hypoglycemia manifestations as (irritability, jitteriness, and convulsions) were done during NICU admission or in the nursery until babies discharged from hospital. Furthermore, BG measurements (Roche HITACHI Cobas C-311 Auto-Analyzer System) were performed at birth, after 30 min, and after 1, 3, 6, 12, 18, and 24 h; follow-up BG evaluations were performed until BG was normalized. We also determined complete blood count (Cell Dyn 3700, automated cell counter, Abbott Diagnostics, USA), electrolytes, CRP, and blood group. Neonatal outcome for neonates admitted to NICU were recorded. Echocardiography study were done before discharge for all IDM newborns met the inclusion criteria and were enrolled in the study.

Approximately 3 mL of UC blood were drawn immediately after delivery from all infants who met the inclusion criteria. The blood was chilled to 4 °C, centrifuged as soon as possible, and stored at − 84 °C. UC serum C-peptide was measured using a third-generation enzyme-linked immunosorbent assay (ELISA) (Modular Analytics E170, Roche Diagnostics, Singapore).

### Data analysis

Data were analyzed using STATA version 14.2 (Stata Statistical Software: Release 14.2 College Station, TX: Stata Corp LP.). Quantitative data were represented as mean, standard deviation, median, and range. Data were subjected to student t-test to compare means of two groups. When the data were not normally distributed, Mann-Whitney’s test was applied. Qualitative data were presented as number and percentage and compared using either the Chi square test or Fisher’s exact test. Graphs were generated using the software packages Excel or STAT; differences were considered significant at a *P* value below 0.05.

## Results

### Patient characteristics

In total, 83 IDM met the inclusion criteria and were included in this study. Of these, 54 (65.06%), developed hypoglycemia and 29 (34.94%) remained normoglycemic. However, there were no significant different maternal or neonatal differences between hypoglycemic and normoglycemic IDMs, even for types of maternal diabetics (*P* value = 0.41), its duration (P value = 0.43), or measurements used for control of diabetes (*P* value = 0.62), as shown in Tables 1 and 2. Furthermore, IDM with hypoglycemia had higher birth weights (3.90 ± 0.81) kg when compared to IDM with normoglycemia (3.78 ± 0.49) kg, although this difference was not statistically significant (*P*-value = 0.07). As regard the echocardiographic finding, ventricular septal hypertrophy (≥ 6 mm) were found in 21 (38.89%) IDM with hypoglycemia compared to 10 (34.48%) IDM with normoglycemia (*P*-value = 0.3).

**Table 1:** Comparison between normoglycemic and hypoglycemic infant according to maternal characteristics

Variable	Normoglycemic *N* = 29	Hypoglycemic *N* = 54	*P* value
Maternal age (years)			
Mean ± SD	36.03 ± 6.93	35.22 ± 4.35	0.51
Maternal weight (kg)			
Mean ± SD	77.76 ± 8.60	79.54 ± 7.90	0.35
Preeclampsia			
Yes	9 (31.03%)	21 (38.89%)	0.18
PROM > 18 h			
Yes	2 (6.89)	4 (7.41%)	0.69
Mode of delivery			
CS	24 (82.75%)	44 (81.48%)	0.25
NVD	5 (17.24%)	10 (18.51%)	
Type of DM			
Gestational DM	12 (41.38%)	23 (42.59%)	0.41
Type 1	0	3 (5.56%)	
Type 2	17 (58.62%)	28 (51.85%)	
Duration of DM (years)			
Mean ± SD	2.21 ± 2.18	3.18 ± 4.3	0.43
Type of treatment of DM			
Diet	1 (3.45%)	5 (9.26%)	0.62
Insulin	23 (79.31%)	40 (74.07%)	
Oral\Insulin	5 (17.24%)	9 (16.67%)

**Table 2:** Comparison between normoglycemic and hypoglycemic infant according to Neonatal characteristics

Variable	Normoglycemic *N* = 29	Hypoglycemic *N* = 54	*P* value
Gestational age (weeks)			
Mean ± SD	38.28 ± 2.59	38.98 ± 2.01	0.11
Gender			
Female	20 (68.97%)	30 (55.56%)	0.23
Male	9 (31.03%)	24 (44.44%)	
Neonatal weight (kg)			
Mean ± SD	3.78 ± 0.49	3.90 ± 0.81	0.07
APGAR score 1 Min			
Median (range)	8 (7–9)	8 (6–9)	0.44
APGAR score 5 Min			
Median (range)	10 (7–10)	10(8–10)	0.45
Hct (%)			
Mean ± SD	53.22 ± 3.59	51.59 ± 4.20	0.08
WBCs (thousands)			
Mean ± SD	11.07 ± 1.81	12.06 ± 3.47	0.16
Platelets (thousands)			
Mean ± SD	221.69 ± 37.10	213.91 ± 45.37	0.43
Serum total Ca (mg/dl)			
Mean ± SD	8.5 ± 0.71	8.32 ± 0.39	0.13
Ventricular septal hypertrophy (≥ 6 mm)			
Number (%)	10 (34.48%)	21 (38.89%)	0.3

### Blood glucose measurements

**Fig. 1:**
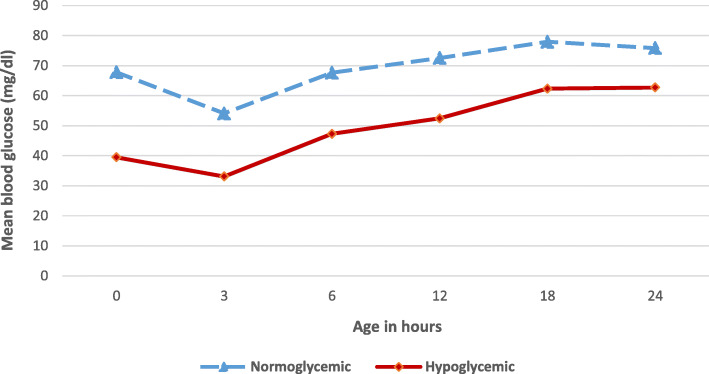
Comparison between normoglycemic and hypoglycemic IDM according to blood glucose during the first 24 h of life

In the hypoglycemic group, the peak of hypoglycemia occurred at the first 3 h of life, with 33.11 ± 8.84 mg/dl for the hypoglycemia group and 54.10 ± mg/dl for the normoglycemic group (*P* = 0.0001; Fig. 1). Furthermore, of a total 54 patients developing hypoglycemia, most of the babies had no hypoglycemic manifestation (96.30%), and only two patients had manifestation one, with lethargy and poor suckling (3.70%).

### Glycated hemoglobin (HbA1C) measurements

**Fig. 2:**
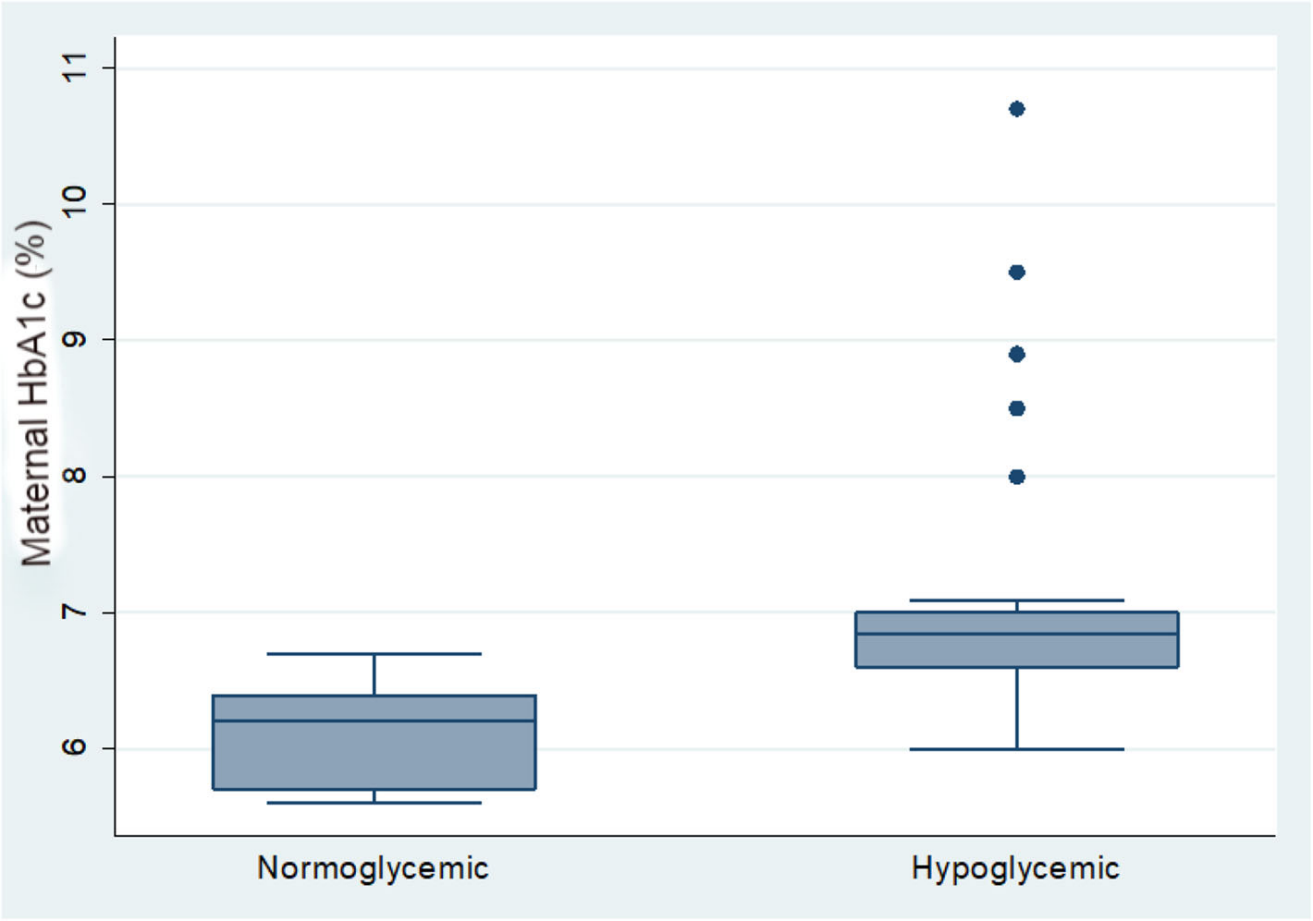
Comparison between normoglycemic and hypoglycemic infant according to maternal HbA1c

As shown in Fig. 2, there were a statistically significant difference between patients developing hypoglycemia and having mothers had poor diabetes control in the last trimester (HbA1C 7.09 ± 0.96%) compared to normoglycemic babies of mothers with good diabetes control (HbA1C 6.11 ± 0.38%), (*P*-value < 0.0001).

### C-peptide measurements

**Fig. 3:**
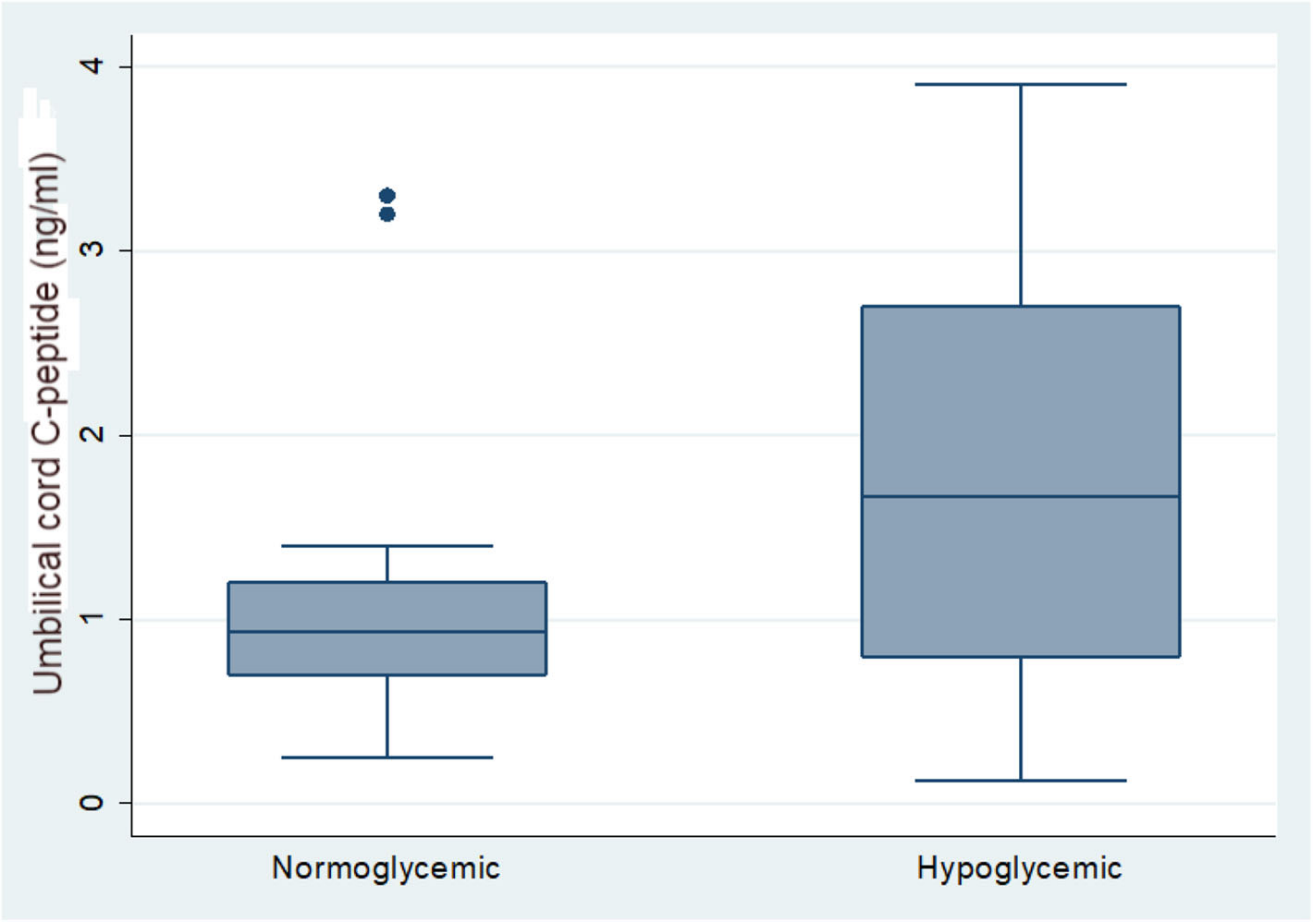
Comparison between normoglycemic and hypoglycemic IDM according serum level of umbilical cord C-peptide

Moreover, as shown in Fig. 3, the mean (SD) of UC C-peptide in the case group was 1.73 ± 1.07 ng/ml, ranging from 0.13 to 3.3 ng/ml, while in the control group, it was 1.08 ± 0.81 ng/ml, ranging from 0.25 to 3.9 ng/ml; there was a statistically significant difference between the two studied groups (*P* value = 0.005).

## Discussion

Our results show that poor diabetes control, especially in the last trimester, is associated with neonatal hypoglycemia. Furthermore, increased UC C-peptide levels could be used as an early indicator for risk of developing neonatal hypoglycemia and a predictor for babies need neonatal admission.

Major risk factors for developing GDM during pregnancy include increased maternal age, a family history of diabetes, a history of GDM in a previous pregnancy, a history of macrosomia in a previous pregnancy, and an increased pre-gravid body mass index [2]. In our study, 35 (42.17%) mothers had GDM, the mean ± SD of maternal age was 36.03 ± 6.93 years, and the mean ± SD of maternal weight was 77.76 ± 8.60 kg.

In this study, at least one attack of hypoglycemia within the first 3 h of life developed in IDM in about 65.06% neonates. This is comparable to the findings of a study by Begum et al. [9], in which the occurrence of hypoglycemia was 73.3% within the first 6 h of life, while in Agrawal et al. [10], only 47% of the infants developed hypoglycemia during the first 2 h of life. In our study, of a total of 54 patients developing hypoglycemia, most cases were asymptomatic hypoglycemia (96.30%), which is in agreement with the findings of previous studies. For example, in a study by Begum et al. [9], about 93.3% of the hypoglycemic babies were asymptomatic, while in Agrawal et al. [10] and Van Howe et al. [11], 100% of the hypoglycemic babies were asymptotic.

Hypoglycemia is the most common metabolic disorder reported in full-term and preterm infants. The definition of hypoglycemia as well as its clinical significance and optimal time at management remain controversial [12]. Previously, asymptomatic hypoglycemia has been considered to be of no clinical significance [13]. However, numerous studies have demonstrated that even asymptomatic hypoglycemia can have a poor neurodevelopmental outcome immediately after birth [14] and even later on up to school age [15]. Therefore, early detection and management of even asymptomatic hypoglycemia are critically important.

In our study, the demographic characteristics of the mothers were similar in hypoglycemic and normoglycemic groups, such as maternal age, maternal weight, and type and duration of diabetes, with similar results when compared to Begum et al. [9]. In contrast, Agarwal et al. [10] who found that IDMs with hypoglycemia had significantly longer durations of maternal diabetes. In our study, infants with hypoglycemia had higher birth weights than normoglycemic babies, although this difference was not statistically significant. This is in agreement with Dawid et al. [16], who found neither a correlation between birth weight and maternal fructosamine level nor between birth weight and maternal HBA1C level. In contrast, Metzger et al. [17] and Cooper et al. [18] found that infants with a higher birth weight were more likely to develop hypoglycemia and hyperinsulinemia than the control group with a normal birth weight, suggesting physiologic relationships between maternal hyperglycemia and fetal insulin production.

We observed a statistically significant difference between infants developing hypoglycemia and having mothers with poor diabetes control in the last trimester and normoglycemic babies. This finding is in agreement with Griffiths et al. [19] and Fallucca et al. [20], who observed a correlation between infant hypoglycemia and poor maternal diabetes control. Poor diabetes control in our cases group mainly related to poor patients compliance and/or resistance against treatment due to lack of regular ant-natal visits as most cases came to our tertiary hospital referred from primary hospitals just before delivery. In contrast, other researchers found that even in well-controlled diabetic mothers, the incidence of early hypoglycemia in infants is still high, particularly in those mothers who had a longer duration of diabetes [10, 16, 21]. Even for some other IDM complications, we found no correlation between the presence of ventricular septal hypertrophy in IDM either with hypoglycemia or normglycemia cases. Other research by Vela-Huerta et al. [22], found no correlation between the increased prevalence of asymmetric septal hypertrophy and the state of maternal diabetic control.

Furthermore, in this study, we found a statistically significant increase in UC C-peptide levels in infants who developed hypoglycemia when compared to the control group (*P* value = 0.005), suggesting that C-peptide can be used as an early predicator for hypoglycemia in IDMs. This finding is comparable with other studies reporting that cord C-peptide levels were inversely related to BG concentrations in the early postnatal period [9, 17, 18, 20]. Furthermore, the increased UC C-peptide level may be associated with infant macrosomia [17, 23] and neonatal septal hypertrophic cardiomyopathy [18]. Therefore hyperinsulemia is the cornstone in the development of many complication in IDM [24]. However, some patients in our case group showing hypoglycemic without elevation of C-Peptide, this points needed to be discussed in further research to study the relation of C- Peptide measurements and hypoglycemia severity.

In conclusion, poor diabetes control, especially in the last trimester, is associated with neonatal hypoglycemia. Furthermore, increased UC C-peptide levels could be used as an early indicator for risk of developing neonatal hypoglycemia and a predictor for babies need neonatal admission. However, further studies with larger sample sizes are needed to determine the cost effectiveness of this relatively costly test before it can be used routinely in daily care practice.

## Data Availability

The datasets used and/or analyzed during the current study are available from the corresponding author on reasonable request.

## Abbreviations

BG: Blood glycose
DM: Diabetes mellitus
GMD: Gestational diabetes mellitus
HbA1C: Glycated hemoglobin
IDM: Infants of diabetic mothers
NICU: Neonatal intensive care unit
PROM: Premature rupture of membranes
UC: Umbilical cord
